# Monitoring modifiable risk factors for breast cancer: an obligation for health professionals

**DOI:** 10.26633/RPSP.2017.80

**Published:** 2017-04-24

**Authors:** Verónica Guerra Guerrero, Antonieta Fazzi Baez, Carmen Gloria Cofré González, Carmen Gloria Miño González

**Affiliations:** 1 Facultad de Ciencias de la Salud Universidad Católica del Maule Talca Chile Facultad de Ciencias de la Salud, Universidad Católica del Maule, Talca, Chile.

**Keywords:** Neoplasms, breast neoplasms, risk factors, life style, primary prevention, Neoplasias, neoplasias de la mama, factores de riesgo, estilo de vida, prevención primaria, Neoplasias, neoplasias da mama, fatores de risco, estilo de vida, prevençãO, primária

## Abstract

*Worldwide, breast cancer is the most common disease in women and constitutes the second leading cause of cancer death in this population. The factors that contribute to the risk of occurrence are divided into nonmodifiable and modifiable factors. Although there are interventions in primary care to prevent the disease, these measures have not produced the desired changes in women’s health. This article reviews the major modifiable risk factors for breast cancer and describes how these factors can affect the incidence of cancer in women. This information shows that modifiable risk factors (such as physical activity, diet, obesity, and use of alcohol and tobacco) can influence the occurrence of breast cancer, in part depending on the life stage of a woman, including menopausal status. Timely prevention at the primary care level is one of the most important areas on which health professionals need to focus in order to help reduce the incidence of breast cancer*.

## SINOPSIS

En todo el mundo, el cáncer de mama es la enfermedad que afecta con más frecuencia a las mujeres y la constituye la segunda causa principal de muerte por cáncer en esta población. Entre los factores responsables del riesgo de cáncer se distinguen los no modificables y los modificables. Aunque hay intervenciones de atención primaria destinadas a prevenir la enfermedad, estas medidas no han logrado los cambios deseados en la salud de la mujer. En este artículo se examinan los principales factores de riesgo de cáncer de mama que son modificables y se describe el modo en que estos factores influyen en la incidencia del cáncer femenino. La información contenida en este artículo demuestra que los factores de riesgo modificables (como la actividad física, la alimentación, la obesidad y el consumo de alcohol y tabaco) determinan la aparición del cáncer de mama y están parcialmente ligados a la fase de la vida en la que se encuentra la mujer, como el estado menopáusico. La prevención oportuna como parte de la atención primaria es una de las áreas más importantes en las que tienen que centrarse los profesionales de la salud para ayudar a reducir la incidencia del cáncer de mama.

## SINOPSE

O câncer de mama é a doença mais comum em mulheres em todo o mundo e constitui a segunda principal causa de morte por câncer nesta população. Os fatores que contribuem para o risco da ocorrência de câncer são divididos em modificáveis e não modificáveis. Embora existam intervenções na atenção primária para prevenir a doença, essas medidas não têm surtido as mudanças pretendidas na saúde da mulher. Este artigo examina os principais fatores de risco modificáveis para o câncer de mama e descreve como esses fatores podem ter efeito na incidência do câncer em mulheres. Os dados indicam que os fatores de risco modificáveis (como atividade física, alimentação, obesidade e uso de álcool e tabaco) podem influenciar a ocorrência do câncer de mama, dependendo em parte do estágio de vida da mulher, inclusive na fase da menopausa. A prevenção oportuna ao nível primário de atenção é uma das áreas mais importantes em que os profissionais da saúde devem se concentrar para reduzir a incidência do câncer de mama.

Globally, breast cancer is the most common cancer among women, representing the second cause of death in this population (1–3). According to a World Health Organization estimate, 410 712 women die annually from this cause worldwide, and there are a total of 1 151 298 new cases per year (4). The increase in the incidence and mortality from this cancer has been attributed to epidemiological changes that are occurring, especially in developing countries (5).

Both the World Health Organization (WHO) (6) and the Pan American Health Organization (PAHO) (7) have declared breast cancer a chronic noncommunicable disease (NCD) that requires prevention and control due to the high economic losses projected for the next 20 years. According to the WHO (8), a large proportion of NCDs are preventable. That is because genetic predisposition or heredity accounts for only 5% to 10% of all cancers (1), while exposure of the population to environmental factors and harmful lifestyles are responsible for the rest (9, 10). In terms of prevention, it has been estimated that 40% of cancers can be averted by reducing risk factors and by primary prevention. In addition, 30% of cancers can be cured with early detection and appropriate treatment (11).

According to PAHO (7), the prevention and control of NCDs, which include breast cancer, requires a holistic approach focused on reducing risk factors and strengthening protective factors. Some studies suggest that prevention needs to occur at the community level, in order to educate women about breast cancer and its treatment. Strategies such as patient education are key for this purpose (1, 12). Educational strategies aimed at lifestyle modification could be crucial to confronting the increased incidence of breast cancer in women (1). Similarly, it is fundamental to strengthen the capacity to monitor and research NCDs, their risk factors, and their determinants (7). Breast cancer, in particular, is curable when detected in early stages (12).

In summary, breast cancer is an NCD that the WHO has declared to be highly preventable by means of changes in modifiable risk factors. Early prevention for breast cancer is crucial among females. This article aims to describe the main modifiable risk factors for breast cancer that require health professionals to develop prevention strategies, mainly at the primary care level.

## MODIFIABLE RISK FACTORS FOR BREAST CANCER

Some studies suggest that not all women have the same risk of breast cancer, and that certain factors, called risk factors, increase the likelihood of developing breast cancer (12). While many of the risk factors are not modifiable, others can be altered (13).

The nonreproductive factors related to lifestyle play an important role in the development of cancer, and they are risk factors that can be modified in the population through behavioral changes (12, 13). These modifiable risk factors include excess body weight, a high body mass index, low physical activity, sedentary lifestyles, excessive alcohol consumption, smoking, and unhealthy diets (12). The modifiable risk factors associated with breast cancer are presented in Table 1 (2, 13–16, 19–24, 27).

### Physical activity

Physical activity is considered to be one of the most important and established modifiable risk factors for breast cancer (14). Physical activity has also been associated with increased life expectancy in the general population and in women who already have breast cancer (15).

Studies show that exercise performed both in adolescence and adulthood helps reduce the risk of developing invasive breast cancer. However, there is no conclusive evidence on the precise age range in which physical activity acts to reduce this risk (14, 16). Nevertheless, it has been reported that physical activity reduces the risk of breast cancer more strongly in postmenopausal women than in premenopausal women (14). Similarly, it has been suggested that physical activity during adolescence is strongly protective against breast cancer (14). For this type of benefit, there is a need for substantial physical activity, on the order of 30 to 60 minutes a day at a moderate-to high-intensity level (14).

**TABLE 1.** Modifiable risk factors associated with breast cancer

Modifiable risk factor	Risk related to breast cancer	Mechanisms related to breast cancer
Physical activity and breast cancer	Low levels of physical activity are associated with a high risk of breast cancer (13). Studies show that an increase in levels of physical activity in women decreases the risk of breast cancer by approximately 20% to 40% (15).	The mechanisms by which physical activity produces a protective effect still remain unclear (16). However, in the general population, these mechanisms have been related to a decrease in exposure to estrogen throughout life and to a decrease in the percent of visceral body fat (13–15). It has been reported that ovarian hormones play a key role in the etiology of breast cancer (14), since physical exercise can reduce the amount of mitogenic hormones available in the body, such as estrogen, insulin-like growth factor, and insulin (16).
Diet and breast cancer	Foods contain different nutrients and compounds that can initiate, accelerate, or reduce the growth of a malignant tumor (20).	The composition of the diet during life, the quality and quantity of unsaturated fat, and the energy balance are all considered factors that contribute independently in the development of mammary gland tumorigenesis, which can lead to breast cancer (19).
Obesity and breast cancer	A strong association between obesity and specific types of cancer (esophageal, pancreatic, colorectal, breast, etc.) has been reported (23).	Regarding the mechanisms underlying the effect of obesity on breast cancer, research results show inconsistencies, finding that these mechanisms depend on the state or degree of the cancer and the timing of diagnosis (23). However, the levels of hormones, such as estrogens, in the body are responsible for facilitating the early development of cancer (24).
Alcohol, tobacco, and breast cancer	In terms of risk, some studies describe a strong link that alcohol and tobacco have with breast cancer. However, other studies have not found conclusive results (2).	The precise mechanism to explain the relationship between alcohol consumption and the risk of disease still remains in dispute, particularly because ethanol, as a chemical by itself, does not have a carcinogenic effect. However, it has been recognized that ethanol produces profound metabolic effects that interfere with the metabolism of other agents (27). There is limited evidence to establish the exact relationship between tobacco and breast cancer.

### Diet

In the general population, diet and physical activity play a fundamental role in preventing breast cancer, and also possibly reduce the risk of such comorbidities as other cancers, cardiovascular disease, and diabetes (17).

In terms of the role of diet in reducing the risk of breast cancer, especially beneficial effects have been found from fruits and vegetables; soy, isoflavones, and lignans; vitamins and minerals; and meats and dairy products (18). The increased consumption of vegetables and fiber is associated with a reduction in total estradiol and plasma estradiol levels, with a decrease in serum estrogen levels, and with an increase in estrogen levels linked to globulin, which influences the bioavailability of estradiol. These estrogen levels are associated with the onset of breast cancer. Regarding soy, isoflavones, and lignans, studies suggest that consumption of these foods is also associated with a reduced risk of breast cancer (19). However, there is no clear evidence to indicate that soy reduces the risk of breast cancer, given that a component of soy can promote the growth of certain tumors that are sensitive to estrogen. Phytoestrogens can act as weak estrogens and as estrogen antagonists, depending on the hormonal status of women (18). Accordingly, for premenopausal women, the increased phytoestrogen intake can compete with endogenous estrogens and reduce the overall estrogen exposure to target tissue. On the other hand, for women with low endogenous levels of estrogens (e.g., postmenopausal women), the phytoestrogens can increase the amount of estrogen activities. Meat consumption, in contrast, has often been associated with an increased risk of breast cancer, although some studies do not support this linkage (5, 18). These divergences could be explained by the different levels of carcinogens and mutagens found in meat, depending on the characteristics in each country. Furthermore, studies suggest that consumption of meat is a factor in the incidence of cancer, as is the meat’s origin and method of preparation (20).

The consumption of dairy products has not been associated with risk of breast cancer. However, the fat content of these products has been described as a promoter of higher cancer risk due to the greater energy intake, resulting in an increased percentage of body fat (18). In contrast, some components present in dairy products, such as calcium and vitamin D, have been associated with a protective effect against breast cancer (18).

Several studies have found a direct association between a healthy diet and a decreased risk for breast cancer. An increased intake of several vitamin and mineral supplements is described as leading to a lower risk of breast cancer; these include carotenoids, folate, calcium, vitamin D, and vitamin C (18). With some other studied foods, such as fish and marine longchain saturated fats, there is controversy as to whether they actually reduce the risk of breast cancer, and the same is true for fat consumption (5).

Using diet to reduce cancer risk is focused on two actions. First, women can increase the consumption of protective foods against cancer, and second, they can reduce foods with carcinogenic effects.

### Obesity

Obesity is associated with the risk of breast cancer. It has been reported that obesity has a dual effect on breast cancer risk. In premenopausal women, there is an inverse association between weight and cancer risk. However, in obese postmenopausal women, there is an increased risk of developing breast cancer (21). Evidence shows that obesity is responsible for about 9% of postmenopausal breast cancer cases. In these cases, the risk of breast cancers increases by 18% for every gain of 5 kg/m^2^ in body mass.

In postmenopausal women, risk is increased due to high levels of circulating estrogens derived from an increase in the conversion of androgens to estrogens by the adipose tissue, and from a high proportion of bioavailable estrogen due to low levels of sex hormone–binding globulin produced by obesity (22). In premenopausal women, obesity is recognized as a protective factor. An explanation for this association could be the greater degree of anovulation in obese premenopausal women, resulting in lower levels of progesterone and estradiol, and in low rates of mammary cell division and decreased cancer risk (22).

The distribution of body fat is described as another influential risk factor for breast cancer. Adipose tissue plays a fundamental role in the body, by means of storing excess calories as fat, and performing endocrine control of other body sites. For example, an increase in central distribution of body mass produces a higher risk of breast cancer in postmenopausal women, particularly when there is a family history of breast cancer (23). The central distribution of body mass, called abdominal obesity or “android obesity,” is a sign of insulin resistance that could be linked to the risk of breast cancer (21).

In summary, breast cancer is characterized by a high prevalence in overweight and obese women, by a high percentage of body fat and central obesity, and by an unbalanced diet (24). Considering that in recent years the prevalence of obesity has increased noticeably in both developed and developing countries, it is essential to develop strategies to prevent this condition and its associated breast cancer risk (10).

### Alcohol and tobacco

Although the relationship between alcohol consumption and cancer varies depending on the type of alcohol, volume, and amount ingested, it has been found that there is a direct relationship. Higher alcohol consumption is associated with an increased risk not only in locations such as the oral cavity, pharynx, esophagus, and larynx, but also with breast cancer. This risk is further increased when associated with tobacco use—a situation warranting special prevention efforts around the world (25).

The relationship between breast cancer and alcohol has been studied in terms of type of alcohol ingested (expressed in millimeters of alcohol present in each drink), age of onset for alcohol consumption, and drinking pattern (regular or occasional) (25). Alcohol consumption is associated with an increased risk of breast cancer, even with moderate consumption and independently of the type of alcohol ingested (5, 26). A study that considered cumulative alcohol intake indicated that there was a 15% (range, 6% to 24%) increase in breast cancer risk even with moderate levels of alcohol intake (5 to 9.9 grams per day or 3 to 6 drinks per week) (27). Another study (5) also suggested that moderate intake of alcohol is associated with an increased risk of breast cancer, and that excessive alcohol intake (30 grams per day) produces adverse effects. The total percentage of breast cancer risk attributable to alcohol has been estimated at up to 5% (27).

Some studies (26, 28) suggest that alcohol increases the risk of breast cancer through five different mechanisms. The first mechanism is that ethanol, which is present in liquor, once ingested, is metabolized to acetaldehyde. Acetaldehyde is a toxic and carcinogenic substance to humans, as it can alter both DNA and body proteins, particularly in breast tissue. The second mechanism is related to the generation of reactive oxygen species, which can damage DNA, proteins, and lipids through an oxidation process. A third mechanism is associated with a deterioration in the body’s ability to dissolve and absorb a variety of nutrients and vitamins, such as B complex nutrients (folate), vitamin C, vitamin D, vitamin E, and carotenoids. The fourth mechanism is associated with increased blood levels of estrogen, which are significantly increased after alcohol ingestion. The fifth mechanism is associated with a reduction in antibody production and the phagocytic capacity of white cells.

With respect to tobacco use, over a long period of time some studies have described some link with breast cancer risk. However, the results are still inconclusive, and there is limited evidence to establish the exact relationship (29). Some studies have found that long-term heavy smoking could be linked with an increased risk of breast cancer. Postmenopausal women who smoke or have smoked are up to 16% more likely to develop breast cancer than are those who have never smoked (30). The risk is also higher in those who started smoking at a young age. Similarly, women who have been heavily exposed to passive smoking, either in childhood or adulthood, may also have an increased risk of developing breast cancer (30).

This evidence on tobacco, however, has been established mainly in laboratory experiments with rodents, whereas in humans the results remain in dispute (30). Nonetheless, the findings indicate that the prevention of smoking, as well as other modifiable risk factors, has a key role in reducing the risk of breast cancer.

## DISCUSSION AND CONCLUSIONS

Breast cancer and other noncommunicable diseases share modifiable behavioral risk factors that are related to lifestyle, including physical inactivity, unhealthy diets, obesity, tobacco, and alcohol. These factors depend on the characteristics of each country and the sociocultural environment around individuals.

Most studies suggest that maintaining a healthy lifestyle that reduces modifiable risk factors is an essential step for reducing the risk of breast cancer. However, there are still no internationally standardized recommendations that would make it possible to define specific prevention models for modifiable risk factors. There are various reasons for that difficulty, including the differences that exist among the women who are at risk of breast cancer. According to the literature reviewed, among recommendations that focus on modifiable risk factors are a holistic approach as well as individual and group education in the community. In addition, it is important to incorporate physical activity and a healthy diet, as well as to reduce excess weight, obesity, and the consumption of alcohol and tobacco. Physical activity recommendations are for 30 to 60 minutes per day, with moderate to high intensity. A healthy diet would include an increase in the intake of vegetables, fruits, and fibers, along with a decrease in the consumption of alcohol, red meat, and animal fat. Reducing obesity and excess weight would focus on the percentage of body fat and central obesity. Finally, daily alcohol and tobacco consumption would be lowered, both in quantity and frequency.

Prevention based on modifiable risk factors for breast cancer needs to be carried out throughout a woman’s life. Prevention should begin at an early stage, but put particular attention on adulthood and the postmenopausal stage. Such risk factors as physical inactivity and obesity require specific prevention efforts. These actions must be appropriate to the life stage of a woman, including taking into account that the risk may take different forms in preand postmenopausal women.

Education as a strategy for preventing cancer risk is applicable both to women with a family history of breast cancer and to women without such a family history but who have modifiable risk factors related to diet, physical activity, obesity, tobacco, or alcohol. Primary care plays an important role in promotion and prevention, by increasing women’s awareness and providing early detection and referral of suspected cases to specialists. Similarly, the prevention of modifiable risk factors through education needs to be incorporated and emphasized in the different levels of general education. These efforts should begin in the early stages of life and should involve the participation of the family and of health professionals.

Existing studies cannot provide conclusive results about the specific relationship between breast cancer and risk factors such as tobacco and alcohol. However, the evidence shows that maintaining a low-fat diet that is rich in fruits and vegetables protects against both breast cancer and other NCDs. To help reduce the risk of cancer in the population, health providers at all levels of care should encourage their patients to regularly engage in physical activity and to reduce excess weight and obesity. Decreasing the risk of breast cancer through prevention of modifiable risk factors is a task that requires the participation of all of society, especially the health professionals who are responsible for the care of the female population.

### Acknowledgments.

The authors wish to thank the Universidad Católica del Maule and the Institutional Improvement Project on Oncology for the support received in the preparation of this article.

### Disclaimer.

Authors hold sole responsibility for the views expressed in the manuscript, which may not necessarily reflect the opinion or policy of the *RPSP/PAJPH* or PAHO.
